# Ventricular Septal Rupture After Blunt Chest Trauma in an Infant: A Case Report and Mini-Review

**DOI:** 10.3389/fped.2020.00316

**Published:** 2020-06-19

**Authors:** Xu Zhu, Xiaojuan Ji, Chun Wu, Harvey Ho, Kunfeng Jiang, Yanqin Wang, Ke Bai

**Affiliations:** ^1^Department of Cardiology, Children's Hospital of Chongqing Medical University, Chongqing, China; ^2^Ministry of Education Key Laboratory of Child Development and Disorders, Chongqing Key Laboratory of Child Infection and Immunity, National Clinical Research Center for Child Health and Disorders, China International Science and Technology Cooperation Base of Child Development and Critical Disorder, Chongqing, China; ^3^Department of Ultrasound, Children's Hospital of Chongqing Medical University, Chongqing, China; ^4^Department of Cardiothoracic Surgery, Children's Hospital of Chongqing Medical University, Chongqing, China; ^5^Auckland Bioengineering Institute, The University of Auckland, Auckland, New Zealand; ^6^Department of Intensive Care Unit, Children's Hospital of Chongqing Medical University, Chongqing, China

**Keywords:** ventricular septal rupture, blunt chest trauma, echocardiography, infant, therapy

## Abstract

Ventricular septal rupture (VSR) due to blunt chest trauma (BCT) is rare in infants. Traumatic VSR should be considered in infants with acute congestive cardiac failure following blunt trauma to the chest. Echocardiography is the method of choice for diagnosis and guiding the management of VSR. In this case report, we present a case of VSR caused by BCT in a 1-year and 9-month-old infant, who was diagnosed by emergency bedside echocardiography. We also provide a mini-review of literatures on BCT-induced VSR in children.

## Introduction

Ventricular septal defect (VSD) is the most common congenital heart disease (1). Acquired VSD is very rare, and is mainly due to trauma, myocardial infarction, or complications of cardiac surgery such as valve replacements or closure of VSD, endocarditis (2). Ventricular septal rupture (VSR), also called traumatic VSD, is a rare complication of blunt chest trauma (BCT) in children (3–5). The formation mechanism of VSD after BCT has been suggested as the ischemic myocardial rupture associated with initial trauma, and/or the reopening of spontaneously closed congenital VSD (6, 7). In addition, during ventricular isovolumic contraction, chest trauma may produce sufficient ventricular force to cause myocardial rupture (6, 7).

Non-invasive imaging of the intra-cardiac structure with high spatial and temporal resolutions can be obtained by echocardiography in children. Therefore, echocardiography is the modality of choice for the diagnosis and management of VSR (4, 8). Bedside echocardiography is the most rapid and feasible modality to diagnose and follow-up acute and severe VSR cases. In this study, we report a case of VSR in an infant following BCT, whereby bedside echocardiography revealed a muscular ventricular septal defect and a ventricular aneurysm on the left ventricular posterior wall opposite the muscular ventricular defect.

## Case

A1-year and 9-month-old male infant was presented with a history of being involved in a motor vehicle accident 5 h earlier in which he sustained blunt force chest trauma. From the time of admission to the 7th day, he was not on a ventilator, and the ECG monitor showed that his vital signs were stable. His breath was in a regular rhythm, and a Grade 2–3/6 systolic murmur was detected at the left lower sternal border. On the 7th day after admission, the vital signs of the infant were unstable: he was in respiratory distress (respiratory rate 70/min), and his heart rate was 145–155 times per minute. His blood oxygen saturation decreased to 78%. Scratches can be seen on the skin of the occipital scalp and the left forearm.

**Figure 1:**
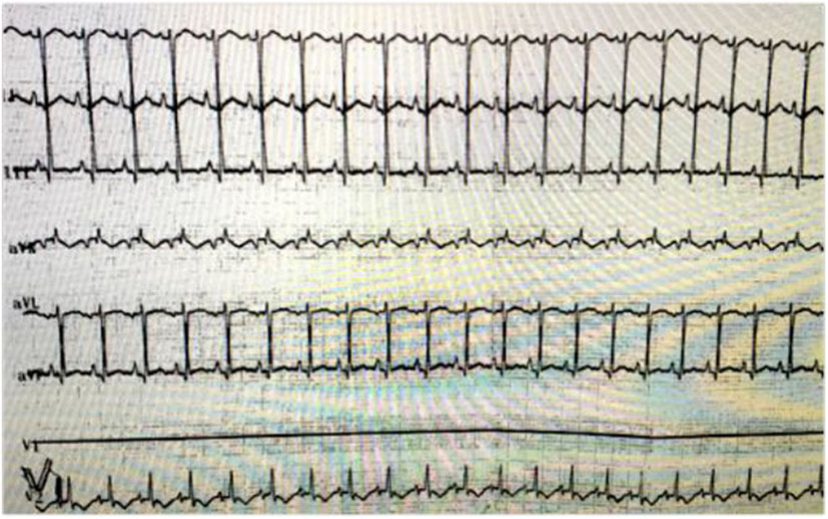
Bedside electrocardiogram revealed ST segment elevation.

Myocardial zymogram evaluations revealed that hypersensitive troponin 1 was increased to 10.281 μg/L, and the creatine kinase MB isoenzyme was increased to 7.29 μg/L. The bedside electrocardiogram showed pathological Q wave and ST segment elevation (Figure 1). Chest computed tomography showed double lung contusion.

**Figure 2:**
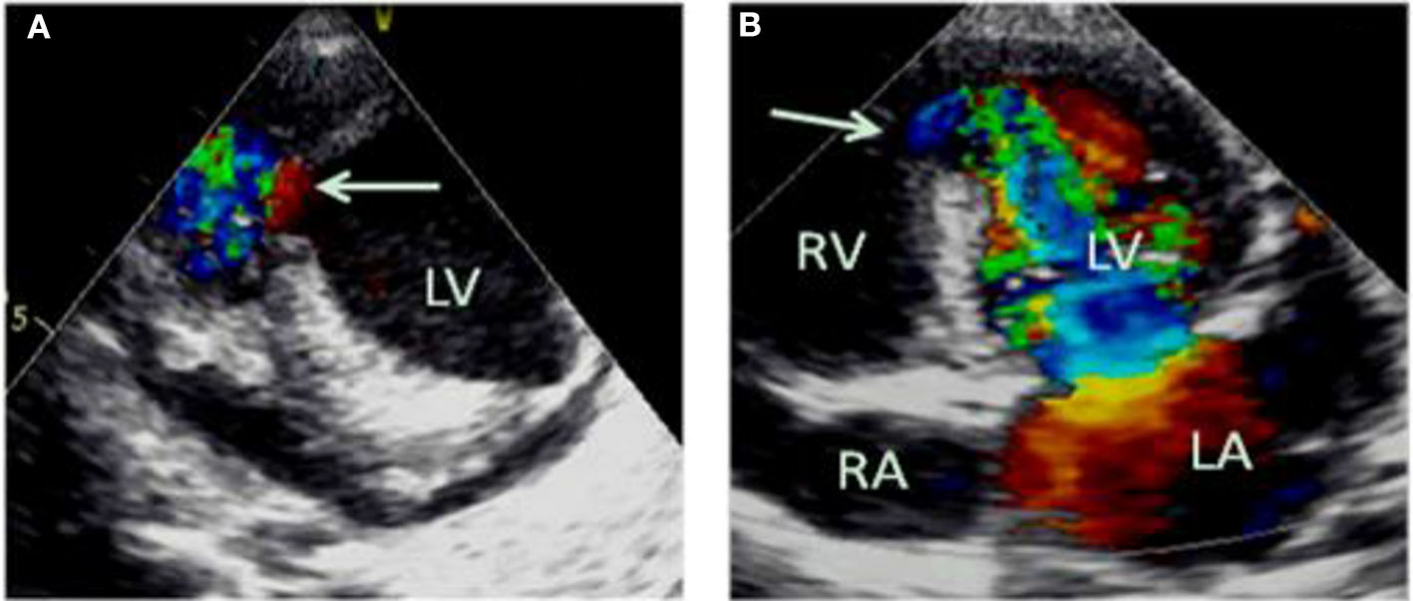
Emergency bedside transthoracic echocardiography revealed funnel shaped M-VSD **(A)**. The white arrows indicate the left and right ventricular shunt orifice **(A,B)**. Color Doppler flow imaging show a bidirectional shunt between the left and right ventricles **(A,B)**. LA, left atrium; RA, right atrium; LV, left ventricle; RV, right ventricle.

**Figure 3:**
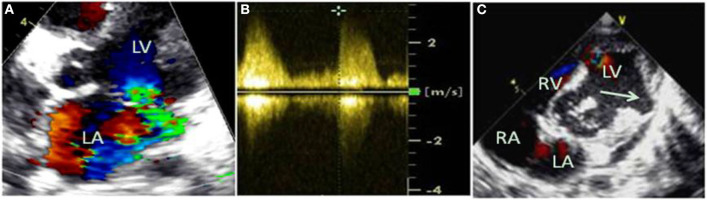
Color Doppler flow imaging revealed severe mitral regurgitation **(A)**, and mild pulmonary hypertension was estimated by continuous-wave doppler **(B)**. Opposite the muscular ventricular defect, a ventricular aneurysm on the left ventricular posterior wall was formed **(C)**, indicated by the arrow.

Emergency bedside echocardiography demonstrated funnel-shaped muscular ventricular septal defect (M-VSD). The diameters of the left and right ventricular shunt orifice were 10 and 5 mm, respectively (Figures 2A,B). Color Doppler flow imaging showed a bidirectional shunt between the left and right ventricles (Figures 2A,B), and also revealed severe mitral regurgitation (Figure 3A), and mild pulmonary hypertension (Figure 3B). Opposite of the muscular ventricular defect, it was noted that a ventricular aneurysm on the left ventricular posterior wall had formed (Figure 3C). The thickness of the myocardium appeared irregular with the thinnest region measuring 3.0 mm.

**Figure 4:**
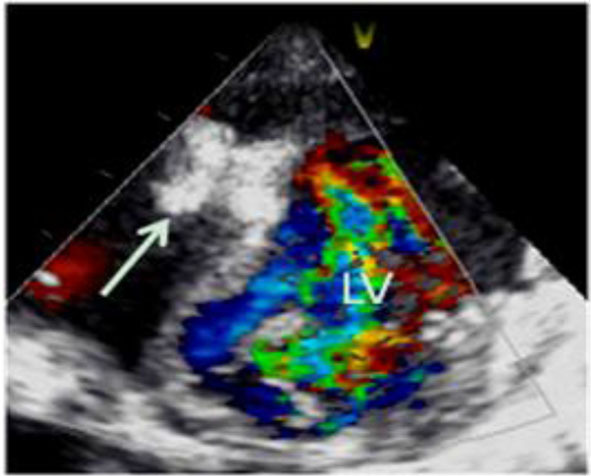
Transthoracic echocardiogram showed no residual shunt after operation, and the arrow indicates the occluding device.

The infant underwent cardio-surgery to occlude the M-VSD with an occluder (SQFDQ-II i, 10 mm, LEPU Medical, China) under direct vision, which was monitored by trans-esophageal echocardiogram. After occlusion of the M-VSD, the ventricular aneurysm on the left ventricular posterior wall was repaired surgically by intermittent pad stitching. The mitral valvuloplasty and foramen ovale suture closure were performed under cardiopulmonary bypass. The intraoperative findings were consistent with those of echocardiography. Both immediate trans-esophageal and trans-thoracic echocardiogram showed no residual shunt after operation (Figure 4).

From post-operation day 1–16 the infant had been in a stable condition (T 36.8°C, R 30/min, HR 131 times per minute, BP 90/54 mmHg, SPO_2_ 96–99%). The cardiac functions of the infant were normal (EF 57% FS 29% E/A 1.4 IRT 42 ms). On post-operation day 13, he was weaned off the ventilator and was extubated. On the 16th day after operation, the blood oxygen saturation rate was decreased (the lowest level reached 27%). The infant was in a critical condition, and tracheal intubation was performed on the patient. His parents decided to give up treatment.

## Discussion

Traumatic VSDs present in 2–10% of BCT cases from motor vehicle accidents, and children are more prone to VSR due to the pliability of the immature chest wall (6, 9, 10). The increased intraventricular pressure after atrioventricular valve closure and the sudden elevation of pressure caused by the impact of BCT make the ventricular septum susceptible to rupture (10–12). The contused myocardium can become necrotic and subsequently perforate because of the two postulated mechanisms of VSR (6, 7). VSR may occur several hours to months after blunt trauma (13). In the case of our infant patient, rupture occurred on the seventh day post-BCT injury.

Congenital VSD usually occurs adjacent to the membranous septum (14). The most common localization of traumatic VSD is in the muscular portion of the interventricular septum near the cardiac apex (12). In this specific case there was no previous history of congenital heart disease. Bedside echocardiography showed M-VSD and a ventricular aneurysm on the left ventricular posterior wall. Electrocardiogram revealed myocardial ischemia, with myocardial zymogram (troponin 1 and creatine kinase MB) further hinting myocardial damage. VSR was diagnosed as a complication of BCT, which was confirmed in the cardiac operation. It is evident in this case study that bedside echocardiography is an effective tool for rapid and accurate assessment of cardiac injury, providing anatomical, and hemodynamic information (8, 15).

The case described herein is similar to the acquired VSR cases due to BCT previously reported in children (4–7, 10). Specifically, Ogunkunle et al. reported BCT induced VSR in a 7-year-old child (4). Behrle et al. reported a case of 6-year-old girl run over by a van, and emergency echocardiograms demonstrated a ventricular septal pseudo-aneurysm (12 × 15 mm) in her superior muscular septum (7). The pseudo-aneurysm was left untreated and remained stable during the patient's 3-month hospitalization. Steed et al. reported a septal avulsion case in a 15-year-old child caused by motor vehicle accident (6), and the aorta of the child was opened in surgery and a septal avulsion was excised through the aortic valve. In another car accident, an anterior M-VSD and a left ventricular aneurysm (28 × 25 mm) were detected in a 13-year-old boy 1 day after the accident (10). The aneurysm was incised during ventricular fibrillation. According to the current literature, our patient (1-year and 9-month old) represents the youngest child whose VSR and septal aneurysm were caused by the complication of BCT after a car accident. A muscular septal occluded device was utilized in a minimally invasive closure, and the aneurysm was also treated in cardiac surgery. Recently, Wu et al. reported a traumatic VSD case in a 1-year-old boy caused by chest compression after a slippery accident by an adult (16). The authors suggest that the appearance of new heart murmur after chest trauma as the clinical clue of traumatic VSD.

## Conclusion

We present the case of a child with VSR following blunt chest trauma due to a motor vehicle accident. The M-VSD was occluded with invasive closure, and ventricular aneurysm was surgically repaired. To our knowledge, similar co-occurrence of these features has never been reported.

## Data Availability Statement

The raw data supporting the conclusions of this article will be made available by the authors, without undue reservation.

## Ethics Statement

Informed consent was obtained from the child' legal guardian for the publication of any potentially identifiable images or data included in this article.

## Author Contributions

XJ, CW, KJ, KB, and YW participated in the diagnosis and treatment of the case. XZ collected clinic data. XZ and HH prepared the manuscript. All authors read and approved the manuscript as submitted.

## Conflict of Interest

The authors declare that the research was conducted in the absence of any commercial or financial relationships that could be construed as a potential conflict of interest.
